# Optothermal Properties of Donor–Acceptor Layers, Including PTB7, PTB7th, Y5, and Y6, for Organic Photovoltaic Cell Applications

**DOI:** 10.3390/ma18081841

**Published:** 2025-04-17

**Authors:** Gabriela Lewinska, Jarosław Kanak, Jerzy Sanetra, Konstanty W. Marszalek

**Affiliations:** 1 Institute of Electronics, AGH University of Krakow, 30 Mickiewicza Ave., 30-059 Krakow, Poland; kanak@agh.edu.pl (J.K.); marszale@agh.edu.pl (K.W.M.); 2 Advanced Diagnostic Equipment Sp z.o.o., Tetmajera Str. 79, 31-352 Krakow, Poland; jsanetra@agh.edu.pl

**Keywords:** morphology, thermal stability, ellipsometry, atomic force microscopy

## Abstract

This study addresses the development and optothermal analysis of donor–acceptor thin layers, including materials universally used in organic photovoltaic cells. This article presents the impact of temperature on the optical properties and morphology of thin films made from materials commonly used in organic solar cells. This research focused on two donor materials (PTB7 and PTB7th) and two non-fullerene acceptors (Y5 and Y6), individually and in binary combinations with PTB7 and PTB7th. This study employed various techniques, including UV–Vis spectroscopy, ellipsometry, and atomic force microscopy (AFM), to analyze changes in the absorption, refractive index, extinction coefficient, and morphology at temperatures ranging from 30 °C to 120 °C. This research shows reversible changes in thickness and absorption with temperature, but the extent of these changes differs between PTB7 and PTB7th. Y5 shows some reversible changes, while Y6 demonstrates greater instability and more permanent changes at higher temperatures. The enhanced thermal stability of binary mixtures compared to single-component materials was observed.

## 1. Introduction

Photovoltaics represent a sustainable and renewable source of energy. Unlike finite fossil fuels, sunlight is an abundant resource that can be harnessed continuously. Global trends can lead to significant strides in achieving carbon reduction goals and mitigating the adverse effects of climate change [1,2,3]. Since the 1950s, successive generations of photovoltaic cells have been developed: mono- and polycrystalline silicon cells [4,5,6] (the first generation), the second generation of thin-film cells [7,8,9], the third generation of dye-sensitized solar cells [10,11,12], organic photovoltaic cells [13,14,15,16], and perovskite cells [17,18] (classified into third or fourth generations). According to Brabec’s triangle [19], the ultimate goal of engineering researchers is to achieve a photovoltaic cell that is highly efficient, has a long life, and is affordable. Lightweight organic solar cells are environmentally friendly and can be deposited on a flexible substrate [20,21,22], allowing for applications in areas where these features are important. They are being intensively researched due to the increasing performance obtained. Recent advancements in organic solar cells have led to significant improvements in power conversion efficiency, reaching up to 20% for devices [23]. Ternary blending and bulk-heterojunction architectures have become viable approaches to improving device performance [21]. Despite these successes, issues with stability, material prices, and long-term dependability still exist [23,24,25].

The commercialization of this new photovoltaic technology must consider the impact of temperature, which is an external factor that we cannot regulate. Photovoltaic cells ultimately operate over a wide temperature range. In the case of silicon solar cells, an increase in temperature reduces the open-circuit voltage, maximum power, fill factor, and efficiency of monocrystalline silicon solar cells [26,27]. Therefore, determining the cause of thermal instability and devising preventative measures for its degradation are crucial. Consequently, intense changes in temperature and the associated optical and morphological properties of the active layer are essential issues in the context of applications [28]. Previous thermal studies on doped layers demonstrated that organic materials are temperature-sensitive [29,30,31,32]. Investigations of the performance of organic photovoltaic cells have revealed the complex effects of temperature. Low temperatures are associated with an increase in the open-circuit voltage and a decrease in the short-circuit current, according to some studies [33], whereas other studies [34] have revealed the reverse trend. Temperature-stable organic cells have also been successfully obtained [35,36,37]. Owing to the complicated photovoltaic process in organic cells (absorption, generation, exciton diffusion and dissociation, transport, and charge collection on electrodes), many elements affect the final cell performance. Research on the various elements of the process is therefore warranted. Di Giacomo et al. [38] demonstrated that lower temperatures reduce charge separation in organic/perovskite photovoltaics, highlighting the importance of entropy in charge generation. Increasing the temperature from 11 °C to 50 °C nearly doubles the electron mobility in organic solar cells, as shown by Zufle [39]. The influence of thermal changes on the optical properties of organic solar cells is critical for improving device performance, stability, and lifespan. Understanding how thermal changes affect the active layer morphology can lead to the development of more controlled and reliable manufacturing processes. This publication contains optothermal studies on thin films of selected organic materials and their mixtures in the context of applications for organic solar cells.

Some of the more popular materials for organic cells today are polymeric donors and small-molecule, non-fullerene donors. This study included popular polymer donors [40] and non-fullerene acceptors [41,42]: PTB7 poly({4,8-bis[(2-ethylhexyl)oxy]benzo[1,2-b:4,5-b′]dithiophene-2,6-diyl}{3-fluoro-2-[(2-ethylhexyl)carbonyl]thieno[3,4-b]thiophenediyl}), PTB7th (poly([2,6′-4,8-di(5-ethylhexylthienyl)benzo[1,2-b;3,3-b]dithiophene]{3-fluoro-2[(2-ethylhexyl)carbonyl]thieno[3,4-b]thiophenediyl})), Y5 (2,2′-((2Z,2′Z)-((12,13-bis(2-ethylhexyl)-3,9-diundecyl-12,13-dihydro[1,2,5]thiadiazolo[3,4e]thieno[2′,3′:4′,5′] thieno[2′,3′:4,5]pyrrolo[3,2-g] thieno[2′,3′:4,5]thieno[3,2-b]indole-2,10-diyl)bis(methanylylidene))bis(3-oxo-2,3-dihydro1H-indene-2,1-diylidene))dimalononitrile) and Y6: (2,2′-((2Z,2′Z)-((12,13-bis(2-ethylhexyl)-3,9-diundecyl-12,13-dihydro-[1,2,5]thiadiazolo[3,4-e] thieno [2′,3′:4′,5′]thieno [2′,3:4,5] [43,44]. PTB7 and its derivative PTB7-Th are low-bandgap polymers that are often utilized in organic solar cells, allowing for power conversion efficiencies of above 9%. These polymers can be used to assess new electron acceptors and interfacial layers, among other elements of device engineering [40,45]. The development of non-fullerene acceptors, particularly Y6 and its derivatives, has shown significantly boosted power conversion efficiency (PCE) beyond 18% [46,47].

The objective of this research is to investigate the optothermal properties of donor–acceptor thin layers commonly used in organic photovoltaic cells. Specifically, this study aims to determine the impact of temperature on the optical properties (absorption, refractive index, extinction coefficient) and morphology of thin films made from PTB7, PTB7th, Y5, and Y6, both individually and in binary combinations. This is performed to understand how temperature affects the performance and stability of these materials in organic solar cell applications and to identify potential strategies for improving their thermal stability. By quantifying the changes in optical properties and morphology, this study will determine the thermal stability of each material and mixture. This helps identify materials more suitable for high-temperature applications. In essence, this research does not merely characterize the materials’ behavior at different temperatures but actively seeks to predict and potentially mitigate the performance degradation in organic solar cells caused by temperature fluctuations. This is crucial for advancing the technology’s reliability and applicability.

## 2. Materials and Methods

**Figure 1:**
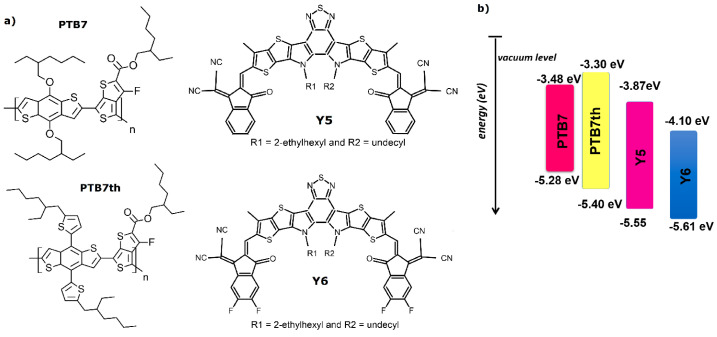
(**a**) Chemical formulas and (**b**) energy diagram of the compounds under investigation [48].

The chemical formulas of the materials under investigation, PTB7, PTB7th, Y5, and Y6, are shown in Figure 1a. Figure 1b shows the energy levels of the materials under consideration. The solutions were made into powder using spectral chloroform. The layers were applied using a VTC-100 vacuum spin coater (MTI Corporation, Richmond, CA, USA) at different speeds, depending on the target layer thicknesses. The layers were applied for atomic force microscopy (AFM) measurements on a glass substrate. For ellipsometry measurements, the layers were applied to crystalline silicon. Absorption measurement was carried out for a specially prepared cell on a quartz substrate. Indium tin oxide (ITO) was applied to part of the cell by radiofrequency spattering around the sample to ensure continuous heating of the sample. ITO was applied in such a way as not to interfere with the absorption measurement (the resulting system was a quartz substrate/measured layer). The voltage source was connected to an ITO semiconductor to provide Joule heating. The ellipsometer would be taken on a thermal stage that provided heating. Temperature was controlled using a thermocouple. AFM measurements were performed before and after the samples were heated in the oven in a vacuum atmosphere.

Layers of pure compounds were investigated to determine their properties. The binary layers that can provide the active layer in the cell were also tested (the donor–acceptor weight ratio was 1:1).

These are the polymer structures used as donor materials in organic solar cells. The chemical structures show how these polymers are built, with PTB7th appearing as a fluorinated version of PTB7, which can enhance certain properties like stability and efficiency.

Y5 and Y6 are non-fullerene acceptors used alongside polymer donors in solar cells. They have different side chains (R1 and R2), which can influence their solubility and interaction with donor materials. Figure 1b provides information about the energy levels of the materials. For PTB7, the HOMO level is localized at −5.28 eV, and the LUMO level was higher than Y6’s value, which was calculated from the difference. PTB7th has slightly a lower HOMO at −5.55 eV. Y5 and Y6 have lower LUMO levels at −3.87 eV and −4.10 eV, respectively.

These energy diagrams help predict how well these materials will perform in electron transfer processes crucial for solar energy conversion.

This paper reports UV–Vis spectroscopy studies performed via an Avantes Sensline Ava-Spec ULS-RSTEC fiber optic spectrophotometer with an Avantes AvaLight DH-S-BAL-Hal lamp (Avantes, Appelsdorn, The Netherlands). AFM research was performed via the Ntegra Aura system in SemiContact mode (NTMDT, Apeldoorn, The Netherlands). An M-2000 ellipsometer (J.A. Woollam, Lincoln, NE, USA) was used for ellipsometric spectroscopy studies. The angles of incidence of the light beam on the sample surfaces were chosen to be 65°, 70°, and 75° for the ellipsometry analyses because of the Brewster angles. The ellipsometric angles delta (Δ is the phase shift between the two vector components of the elliptically polarized light) and psi (Ψ is the amplitude attenuation ratio of the two components) were used to quantify the change as the polarization state of p- and s-polarized light changed. AFM measurements were conducted before and after heating for 20 min at 110 °C. Absorption was measured using a UV-Vis spectrometer, while the results of refractive indices, extinction coefficients, and thicknesses were determined based on ellipsometry.

## 3. Results and Discussion

### 3.1. Single-Compound Thermal Stability of Donors: PTB7 and PTB7th

**Figure 2:**
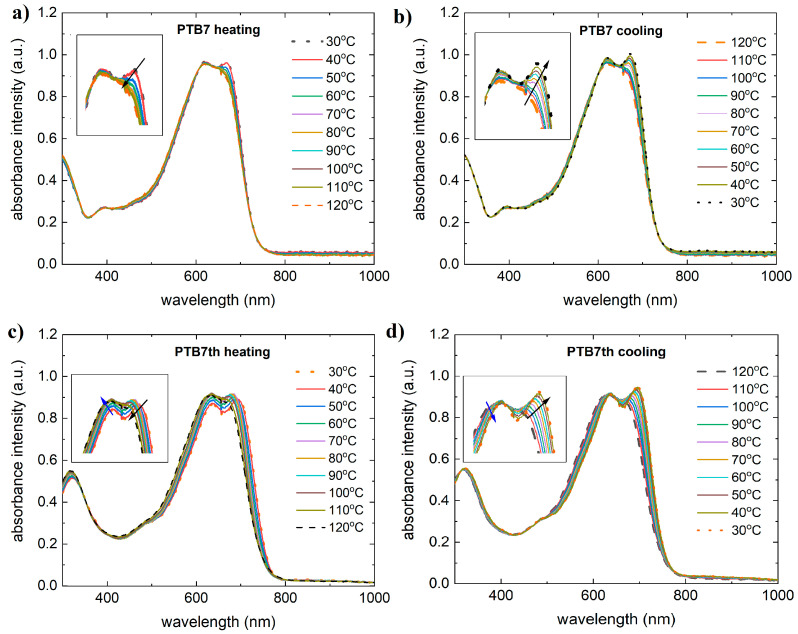
Absorbance spectra of the investigated donors: (**a**) PTB7 during heating, (**b**) PTB7 during cooling, (**c**) PTB7 during heating, and (**d**) PTB7 during cooling. Arrows on the figure indicate the direction of changes.

The thin film absorption spectra of the pristine donors at different temperatures are shown in Figure 2. The absorption spectrum of PTB7 shows a broad band from 500 nm to 700 nm, with two maxima: 630 nm and 680 nm. As annealing decreases the absorption intensity, the 630 nm peak fades above 100 °C. However, this is reversible; for PTB7th, the peak increases in intensity during cooling. The PTB7th thin film shows absorption maxima which, during heating, for the 630 nm peak, the intensity increases, and for the other one, it decreases. This process is reversible during cooling.

Thermal changes in PTB7 and PTB7th were observed by Fernandes et al. [43] via complementary measurements via nuclear magnetic resonance spectroscopy methods (total mass percentage, 1H nuclear magnetic resonance spectroscopy).

**Figure 3:**
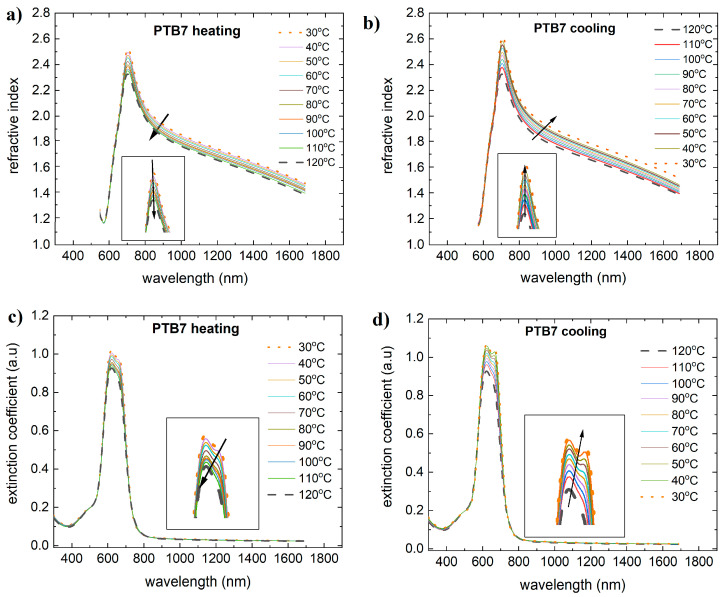
Temperature changes as a function of wavelength for (**a**) the refractive index (heating), (**b**) the refractive index (cooling), (**c**) the extinction coefficient (heating), and (**d**) the extinction coefficient (cooling) for PTB7. Arrows on the figure indicate the direction of changes.

The dispersion dependence of the refractive index and extinction coefficient on temperature for PTB7 is shown in Figure 3. Temperature variations in the extinction coefficient will result in maxima in the absorption band. These maxima, as in the case of absorption, decrease in magnitude. The refractive index in the absorption band increases up to 700 nm and then decreases. For PTB7, during heating and cooling, there is an increase in the refractive index in a part of the band (450 nm to 660 nm). The maximum refractive index shows a reversible change, decreasing during heating and increasing during cooling.

Spectroscopic ellipsometry provides relevant information on the electronic and optical properties of the samples. The models were fitted to the experimental data (psi Ψ and delta Δ ellipsometry angles) via software associated with ellipsometry hardware. The complex dielectric coefficients in this model are related to the complex refractive index. In the $\tilde{N}$ dispersion relation (Equation (1)), *k* is the extinction coefficient, and n is the refractive index that determines the phase velocity.

(1) $$\tilde{N}=n+ik$$

After considering an appropriate substrate for the layers under investigation, refractive indices and extinction coefficients were obtained through the Genosc™ parameterized semiconductor oscillator functions [49] (derivatives of Lorenz functions). The optical characteristics of high extinction coefficient films must be precisely described by the models. For the films under consideration, the Tauc–Lorentz [50] (Equation (1)) and Cody–Lorentz [51] (Equation (2)) models were selected.

(2) $$\varepsilon_{2\;Tauc-Lorentz}\;\left(E\right)=T\left(E\right)L\left(E\right)=\frac{1}{E}\;\frac{A\;E_{0}C{\left(E-E_{g}\right)}^{2}}{{\left(E^{2}-E_{0}^{2}\right)}^{2}+C^{2}E^{2}}$$

In Equation (2), parameter *A* is the transition amplitude, *C* is the broadening coefficient of the Lorentz oscillator, *E*_0_ is the peak position of the Lorentz oscillator, *E* is the photon energy, and *E_g_* is the bandgap energy.

The combination of the Lorentz oscillator function *L*(*E*) and the variable band-edge function *G*(*E*) yields $\varepsilon_{\;2Cody-Lorentz}$ when the photon energy is greater than *E_t_*.

(3) $$\varepsilon_{2\;Cody-Lorentz}=\left\{\begin{array}{c} if\;0<E\leq E_{t}\;\frac{E_{t}G\left(E_{t}\right)L\left(E_{t}\right)}{E}\;\exp(\frac{E-E_{t}}{E_{U}})\\ if\;E_{t}<E\;G\left(E\right)L\left(E\right) \end{array}\right.$$

In Equation (3), *G*(*E*) is the variable band-edge function, *L*(*E*) is the Lorentz oscillator function, *E_u_* determines the rate at which the Urbach tail decreases with decreasing photon energy, *E_t_* determines where the Urbach tail starts [52]. The models are consistent with the Kramers–Kroning relation, which uses ${\;\varepsilon }_{1\;Cody-Lorentz}$ and $\varepsilon_{1\;Tauc-Lorentz}$.

**Figure 4:**
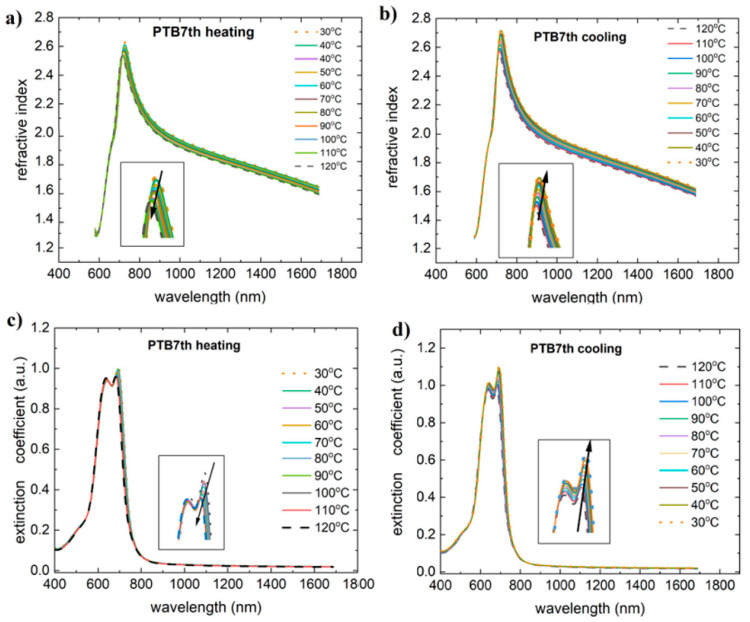
Temperature changes as a function of wavelength for the (**a**) refractive index (heating), (**b**) refractive index (cooling), (**c**) extinction coefficient (heating), and (**d**) extinction coefficient (cooling) for PTB7th. Arrows on the figure indicate the direction of changes.

PTB7th also shows similar behavior in terms of the dispersion dependence of the refractive index (Figure 4). The extinction coefficient also has two maxima for PTB7. In the case of the PTB7th donor, refractive index maxima first decrease during heating and then increase during cooling. Analogous changes during heating and cooling are observed for extinction coefficients.

**Figure 5:**
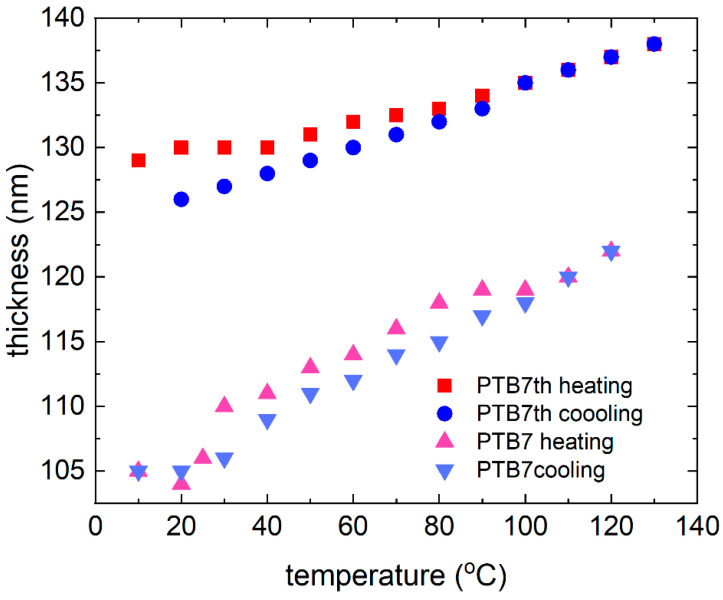
Thin film thickness dependence for donors: PTB7 and PTB7th during the heating and cooling processes.

The temperature dependence of the pristine PTB7 and PTB7th thin film thicknesses during heating and chilling is depicted in Figure 5. The size of the symbol can be considered the uncertainty of determining the thickness. The donor layers show changes in the thickness of the layer. The donor polymers PTB7 and PTBth show reversible changes in thickness and absorption with temperature, but the extent of these changes differs between PTB7 and PTB7th, indicating sensitivity to subtle structural differences (in PTB7th, thiophene has been incorporated into the benzene ring instead of oxygen, which is in PTB7). For a thin layer of pure PTB7th, there is a linear increase in layer thickness of approximately 8 nm (approximately 7%). For the PTB7th layer, a significant increase in the layer thickness of approximately 15 nm (14%) is observed. These changes are reversible, and most materials expand upon heating and contract upon cooling. In polymer films, this affects the intermolecular spacing and packing density [53,54]. Packing variations impact the degree of π–π stacking (in the case of conjugated polymers) or other intermolecular interactions that impact the intensity of absorption. The peak intensity typically increases with closer packing and decreases with greater separation. Changes in the absorption and extinction coefficients and refractive indices are associated with thermal broadening. Molecules, especially more complex ones such as polymers, have multiple energy levels associated with vibrations and rotations, making their absorption spectra more stretched and forming broad bands instead of single lines. These bands are formed by the overlap of multiple energy transitions associated with different modes of vibration and rotation in the molecule. Temperature affects these vibrations and rotations, leading to additional blurring of the bands [55].

**Figure 6:**
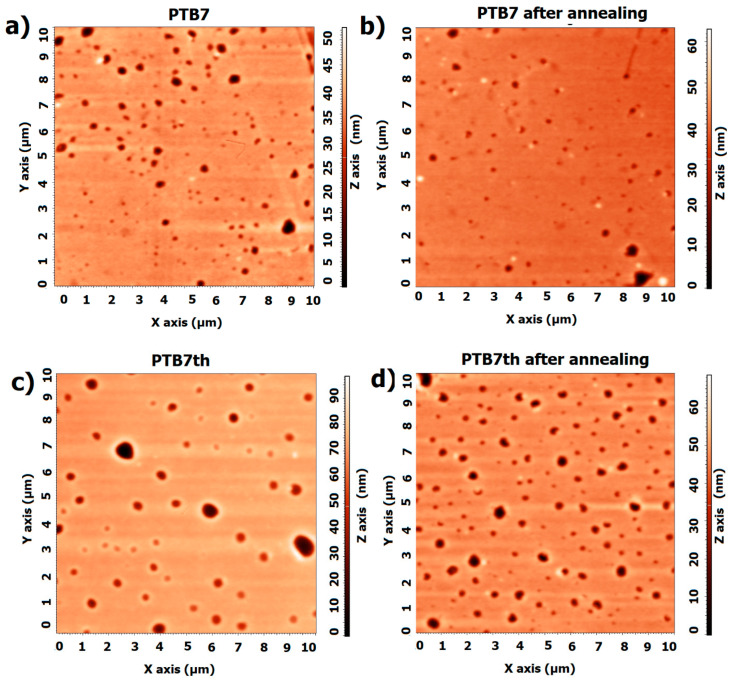
AFM images of thin films (**a**) PTB7 before annealing, (**b**) PTB7 after annealing, (**c**) PTB7th before annealing, and (**d**) PTB7th after annealing.

**Figure A1:**
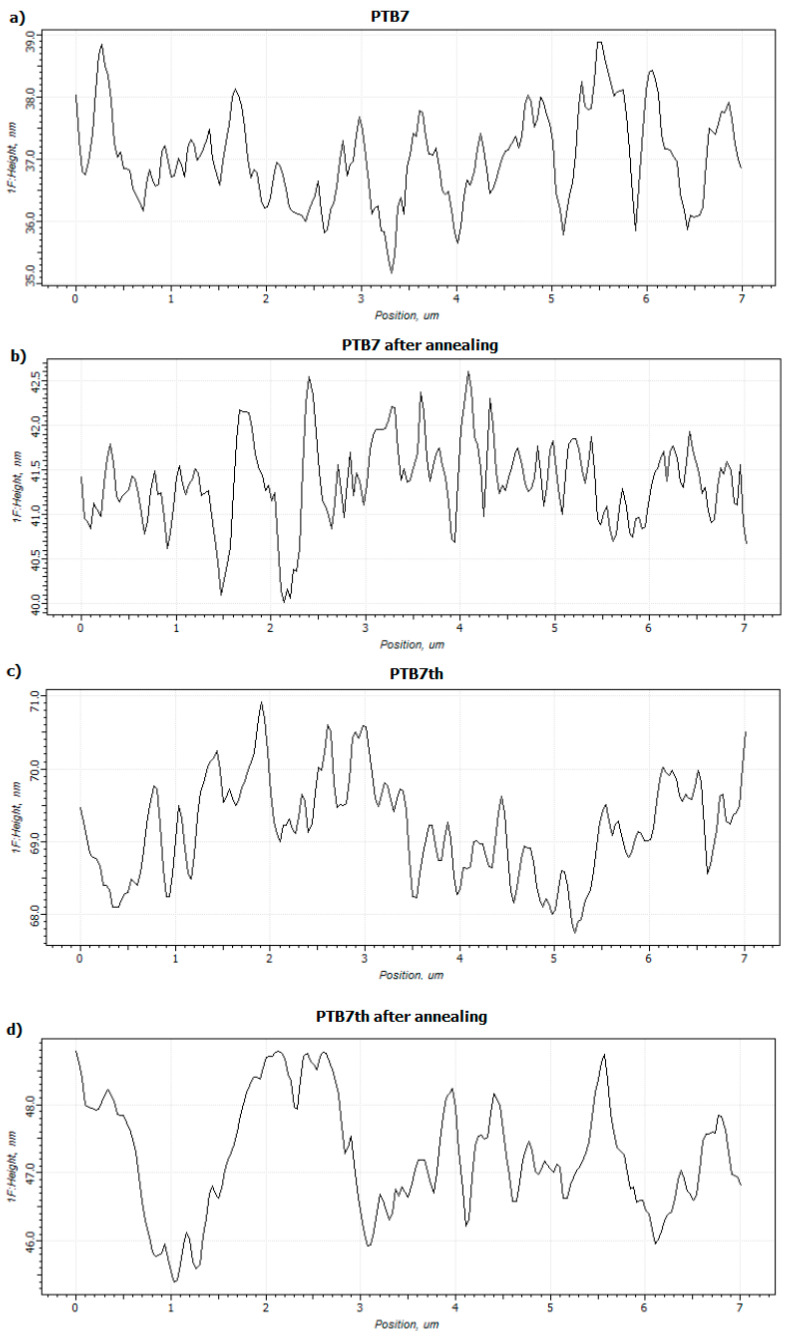
AFM height profile for (**a**) PTB7 before heating (**b**) PTB7 after annealing and (**c**) PTB7th before and (**d**) after annealing.

AFM measurements were performed to check for changes in the morphology of the surface. As shown in Figure 6, the pure layers of both PTB7 and PTB7th do not significantly change in structure. However, there is a slight difference in the values of root mean square roughness Rq and average roughness Ra, which for PTB7 are Ra: 3.5 nm, Rq: 1.7 nm before annealing and Ra: 2.36 nm, Rq: 0.99 nm after annealing. For PTB7th, the Ra is 6.7 nm, Rq: 2.7 nm before annealing, and after annealing, the Ra is 5.1 nm, Rq: 2.5 nm. These differences are of the order of magnitude of measurement uncertainty. Similar changes can also be observed in the AFM profiles (Appendix A, Figure A1).

High-temperature annealing processes lead to the fragmentation of alkyl side chains in various conjugated polymers [56], but moderate-temperature annealing for PTB7 results in chains that rearrange into a more ordered configuration [57]. These chemical changes are responsible for changes in the absorption peaks and refractive indices and extinction coefficient dispersion spectra for both donors. A thickness reduction following 290 °C annealing was confirmed via profilometry (approximately 15%). The loss of side chains for both PTB7 and PTB7th was shown by Fernandes et al. [43]. The thiophene side chains in PTB7th show greater thermal resistance than the ether chains in PTB7. This is confirmed by greater changes in PTB7 than in PTB7th.

### 3.2. Single-Compound Thermal Stability of Non-Fullerene Acceptors Y5 and Y6

**Figure 7:**
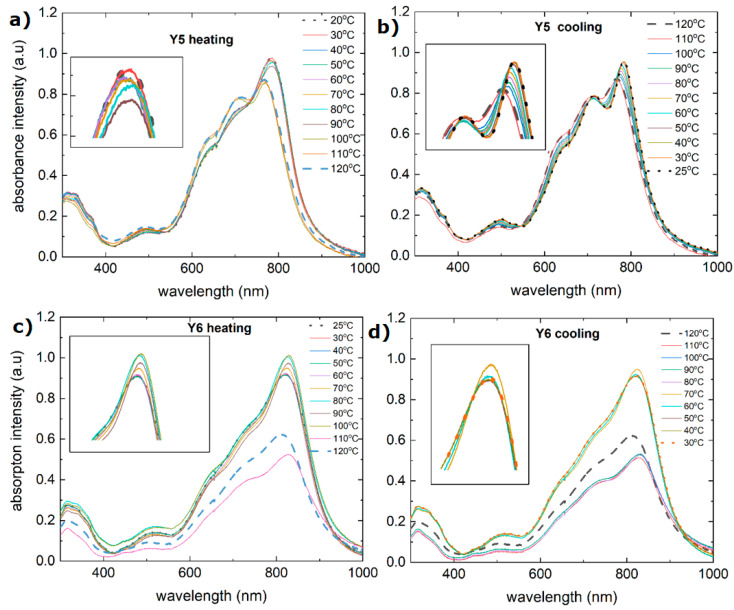
Absorbance spectra before and after heating for the investigated acceptors: (**a**) Y5 during heating, (**b**) Y5 during cooling, (**c**) Y6 during heating, and (**d**) Y6 during cooling.

The absorption spectra of the non-fullerene acceptors Y5 and Y6 are shown in Figure 7a,b. The absorption spectrum of the Y5 thin film consists of a band from 300 nm to 400 nm and a broad band from 420 nm to 900 nm, with maxima at 490 nm, 630 nm, 700 nm, and 800 nm (at room temperature). The absorption spectrum of the Y6 compound also has a band with a maximum at 330 nm and a similar band from 400 nm to 980 nm but with less distinct maxima at 500 nm, 650 nm, 700 nm, and 810 nm (at room temperature).

**Figure 8:**
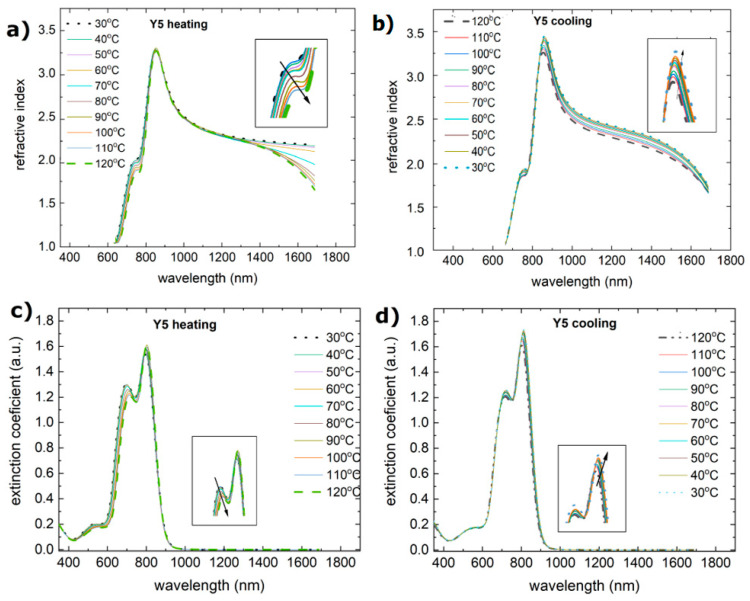
Temperature changes as a function of wavelength for the (**a**) refractive index (heating), (**b**) refractive index (cooling), (**c**) extinction coefficient (heating), and (**d**) extinction coefficient (cooling) for Y5.

During the heating process, the Y5 layer exhibited a change in absorbance at approximately 110 °C. There is a shift in the maximum from 810 nm to 800 nm, and the maxima at 650 nm and 700 nm become more pronounced. There was also a decrease in the absorbance intensity. Similar changes are observed for the extinction coefficient and refractive index (Figure 8). The process of absorbance is reversible, and the intensity of the absorbance returns to the initial state. However, both the refractive index and extinction coefficient decrease permanently. It is connected with the glass transition reported at 102 °C [58].

The lack of change in the refractive indices and extinction coefficients confirms the stability results shown by the absorption spectra.

The absorption spectrum (Figure 7c,d) of Y6 has a similar bandwidth to that of Y5, with some shifted maxima at 360 nm, 510 nm, 630 nm, 710 nm, and 820 nm. During heating, the absorption intensity initially increases slightly with temperature and then decreases at 110 °C. The size of the symbol can be considered the uncertainty of determining the thickness. This indicates that while some thermal energy initially enhances the absorption by improving the molecular alignment, further heating could cause structural changes or degradation that reduce the absorption. The absorption spectrum during cooling appears to recover to some extent, although not perfectly. This suggests that some changes due to heating may be reversible but not entirely reversible. Interestingly, both the dispersion relation and the refractive index do not degrade with increasing temperature. Changes can be observed only in the infrared region, although this is probably due to the uncertainty of model fitting.

**Figure 9:**
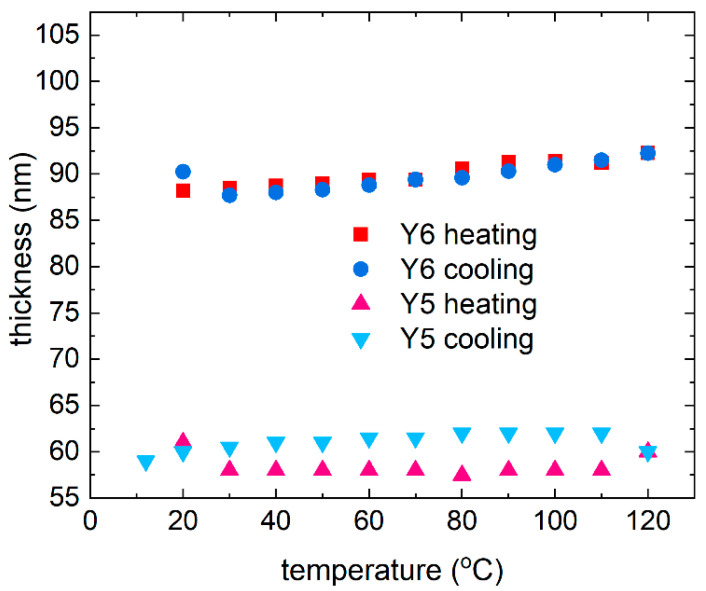
Thin film thickness dependence of the Y5 and Y6 acceptors during the heating and cooling processes.

Similar dispersion relations for Y6 (for room temperature) were obtained by Kerremans et al. [59]. The temperature dependence of the thickness of the pristine Y5 and Y6 thin films is shown in Figure 9. Thin layers of single NFA compounds show slight changes in thickness (7 nm for Y5 and 3 nm for Y6) during heating and cooling cycles. During heating, Y5 is noted to increase slightly in thickness upon heating.

**Figure 10:**
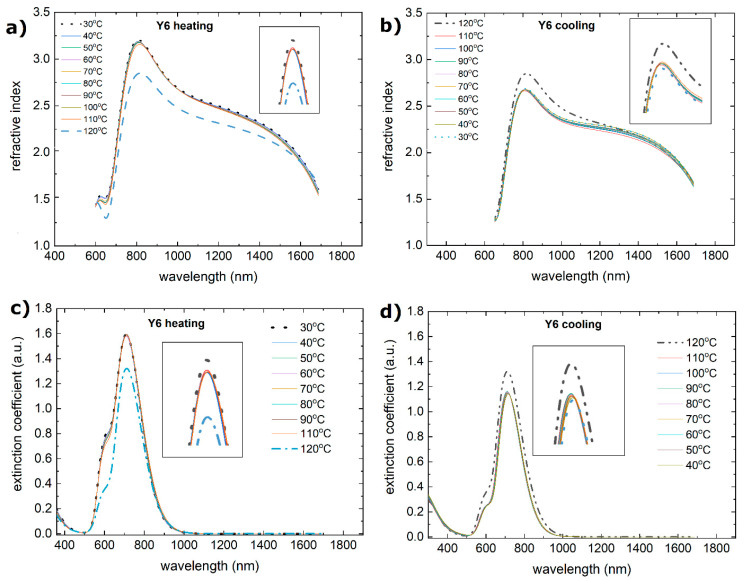
Temperature changes as a function of wavelength for the (**a**) refractive index (heating), (**b**) refractive index (cooling), (**c**) extinction coefficient (heating), and (**d**) extinction coefficient (cooling) for Y6.

**Figure 11:**
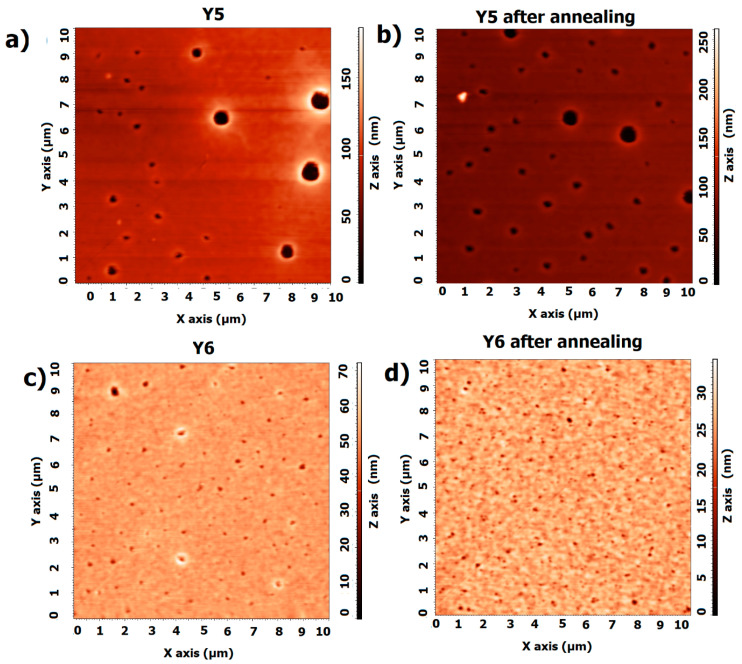
AFM images of thin films (**a**) Y5 before annealing, (**b**) Y5 after annealing, (**c**) Y6 before annealing, and (**d**) Y6 after annealing.

The thickness returns to near initial values during cooling, suggesting that the material has the ability to regenerate after the thermal cycle. For Y6, the thickness remains relatively constant at temperatures up to 120 °C, indicating stability during heating; during cooling, it also remains constant, as it does during heating, suggesting that the structure does not change significantly. For crystalline materials, changes in structure due to heating (e.g., melting, recrystallization) can lead to permanent changes in the material’s optics. We also expect that heating can affect the distribution of impurities or defects in the material, which also affects its optical properties. However, the changes in Y5 and Y6 are reversible and minor. Figure 10 shows the heating and cooling changes for non-fullerene acceptors. The AFM images of the Y5 thin films (Figure 11) show a slight increase in height variation, which might indicate that annealing caused some rearrangement or growth of the material.

**Figure A2:**
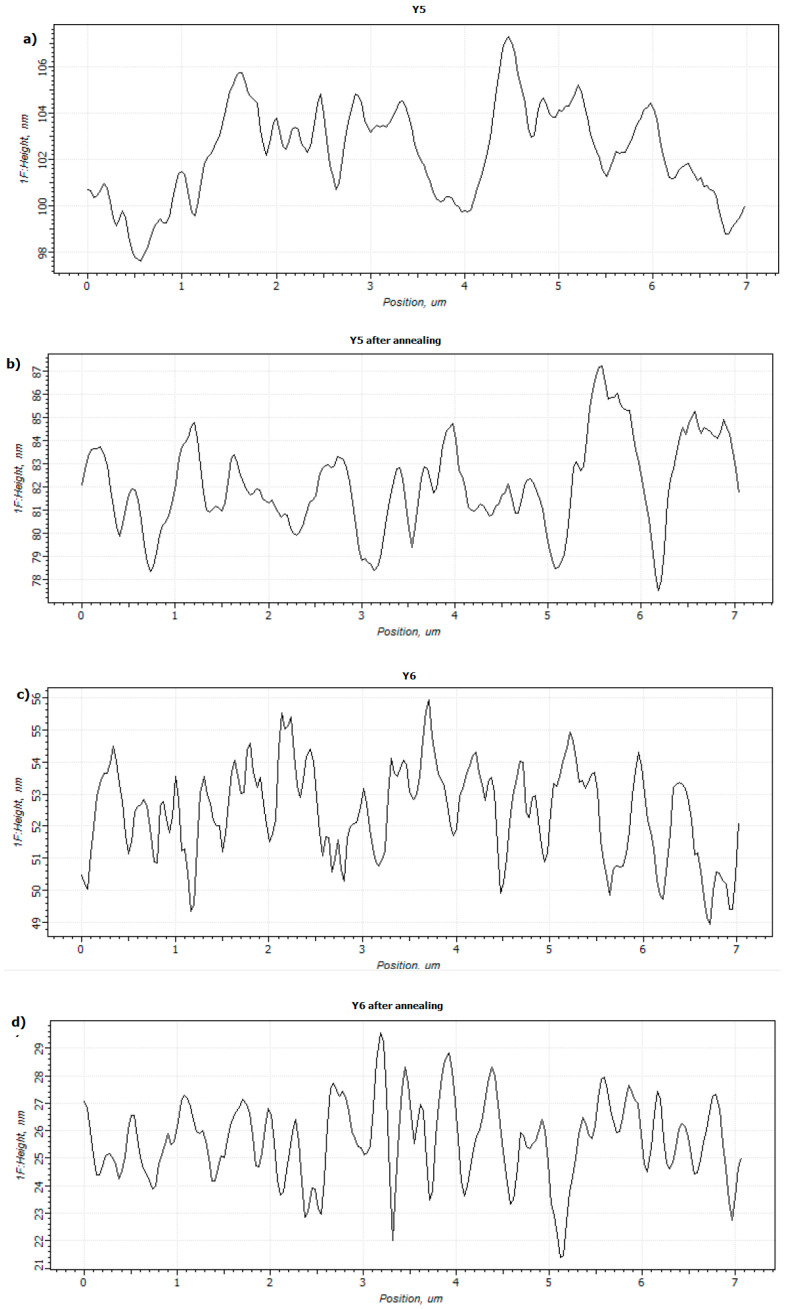
AFM height profile for (**a**) Y5 before heating, (**b**) Y5 after annealing, and (**c**) Y6 before and (**d**) after annealing.

For Y6, the image before annealing shows a relatively smooth surface with some distinct circular features that appear as depressions or holes. These features likely represent regions where the material is thinner or less dense. In terms of scale, the changes in roughness are not significant. The AFM profiles are displayed in the supporting documentation (Appendix A, Figure A2).

For the Y5 layer, Rq decreases from 11 nm to 9.4 nm after annealing, and Ra decreases from 5.5 nm to 3.8 nm. For the Y5 film, a reduction in the roughness of the layer is observed. For the pure Y6 layer, there is also a reduction in roughness (Rq from 2.5 nm to 2.0 nm, Ra from 1.6 nm to 1.5 nm). The NFA materials are sensitive to temperature changes; however, the processes appear only above 100 °C.

Despite the thermal instability of Y6 above 100 °C, it was not ruled out because of the improved compound stability with blending [60,61].

### 3.3. Binary Mixtures Based on PTB7 Donor Thermal Stability

An analysis of the thermal properties of binary mixtures was carried out. They were categorized according to which donor was the base of the mixture.

**Figure 12:**
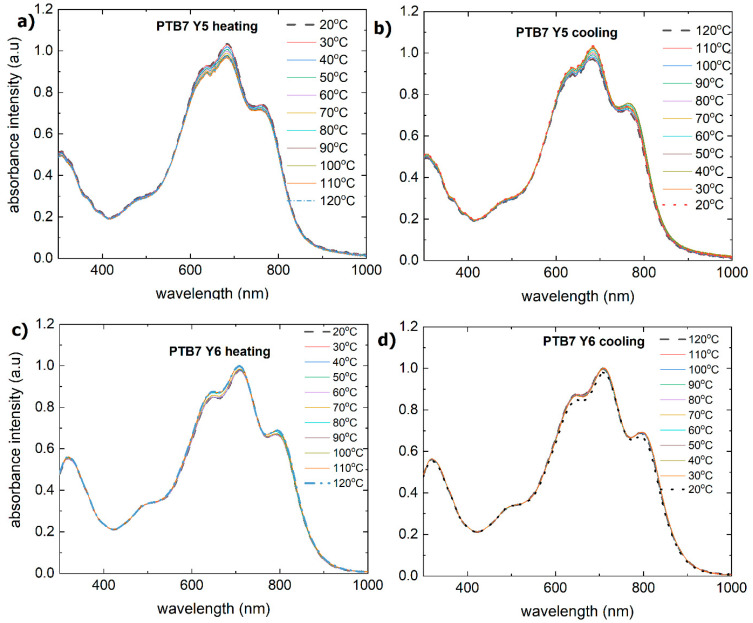
Absorbance spectra of the investigated binary thin films: (**a**) PTB7:Y5 during heating, (**b**) PTB7:Y5 during cooling, (**c**) PTB7:Y6 during heating, and (**d**) PTB7:Y6 during cooling.

The PTB7:Y5 thin film has a broad absorption spectrum (Figure 12a,b) with peaks at 330 nm, small peaks at 385 nm and 390 nm, and clear peaks from the following main bands: 480 nm, 610 nm, 670 nm, and 780 nm. During heating, the intensities of the 610 nm, 670 nm, and 780 nm maxima increase slightly and decrease during cooling.

**Figure 13:**
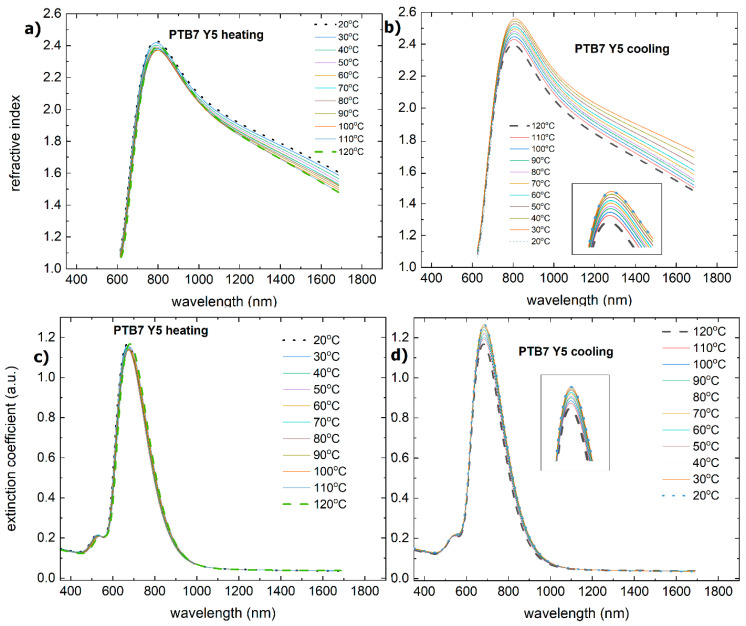
Temperature changes as a function of wavelength for (**a**) the refractive index (heating), (**b**) the refractive index (cooling), (**c**) the extinction coefficient (heating), and (**d**) the extinction coefficient (cooling) for the PTB7:Y5 mixture.

No change in the absorption spectrum is observed for this layer, indicating that the system is thermally stable. Figure 13 shows that the extinction coefficient during heating is constant but slightly increases during cooling (from 1.1 to 1.2). 

Up to a wavelength of 600 nm, there is no change in the refractive index during temperature changes. During cooling, the maximum changes (increases from 2.4 to 2.6 for wavelengths of 780 nm). There is also an observed increase in the refractive index for wavelengths up to 1600 nm. This phenomenon occurs because the local density of the film increases, which can contribute to an increase in the refractive index.

The spectra of binary mixtures show slightly more peaks than the spectra of layers based on single compounds. The difference between the peak positions is at the level of 100 nm, which in the 800 nm region corresponds to an energy of about 0.1 eV. This difference is most likely related to transitions to different oscillation levels.

**Figure 14:**
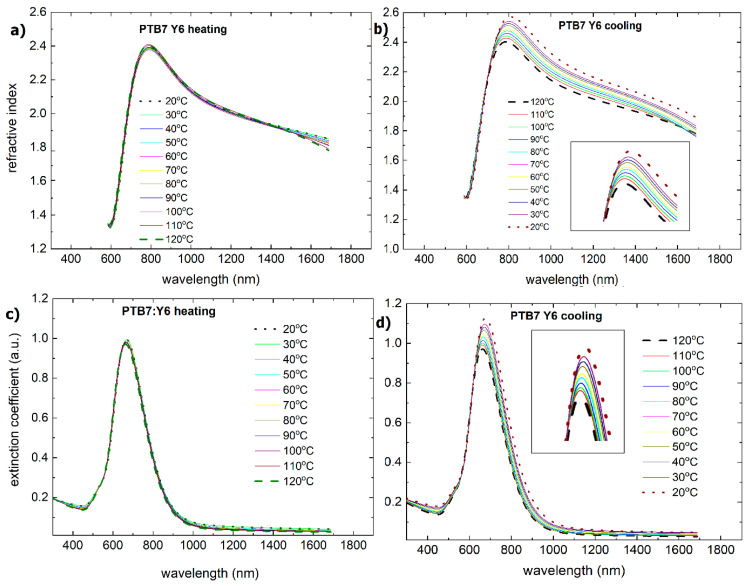
Temperature changes as a function of wavelength for (**a**) the refractive index (heating), (**b**) the refractive index (cooling), (**c**) the extinction coefficient (heating), and (**d**) the extinction coefficient (cooling) for the PTB7:Y6 mixture.

The PTB7:Y6 thin film also has wide absorption bands and peaks at similar positions: 350 nm, 490 nm, 620 nm, 700 nm, and 800 nm (Figure 12c,d). The dispersion characteristics of the refractive indices and extinction coefficient for PTB7:Y6 (Figure 14) are similar to those of the PTB7:Y5 layer: they are stable during heating and change from 620 nm to 1600 nm during cooling (increase).

**Figure 15:**
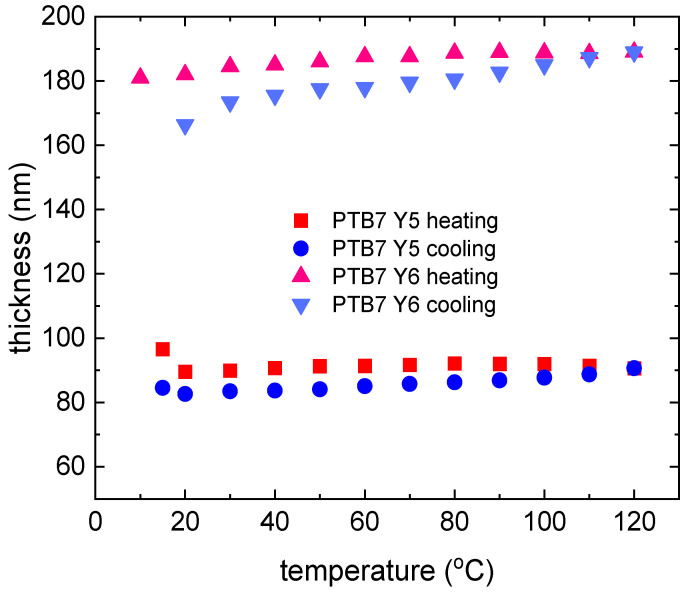
Thin film thickness dependence of the PTB7:Y5 and PTB7:Y6 thin film mixtures during the heating and cooling processes.

The thickness changes (Figure 15) in the layers are approximately 10 nm; during heating, there is a slight increase for PTB7 and then a decrease in the thickness for PTB7:Y5. The thickness of PTB7:Y5 with heating does not decrease but slightly increases during the heating process. A slight increase can be observed at approximately 20 °C. The symbol’s size is taken into account while estimating its thickness. The thickness of the thin film decreases (Figure 15) by approximately 10%.

**Figure 16:**
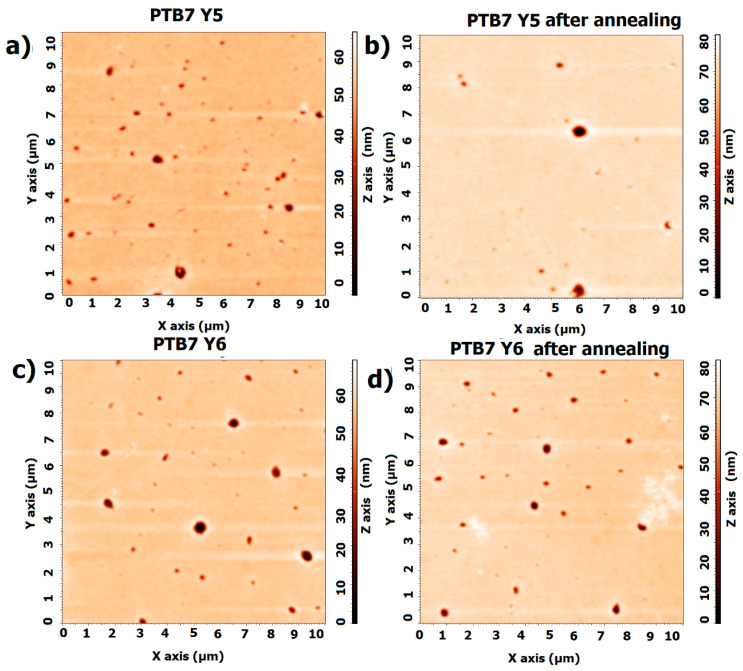
AFM images of thin films of (**a**) PTB7:Y5 before annealing, (**b**) PTB7:Y5 after annealing, (**c**) PTB7:Y6 before annealing, and (**d**) PTB7:Y6 after annealing.

**Figure A3:**
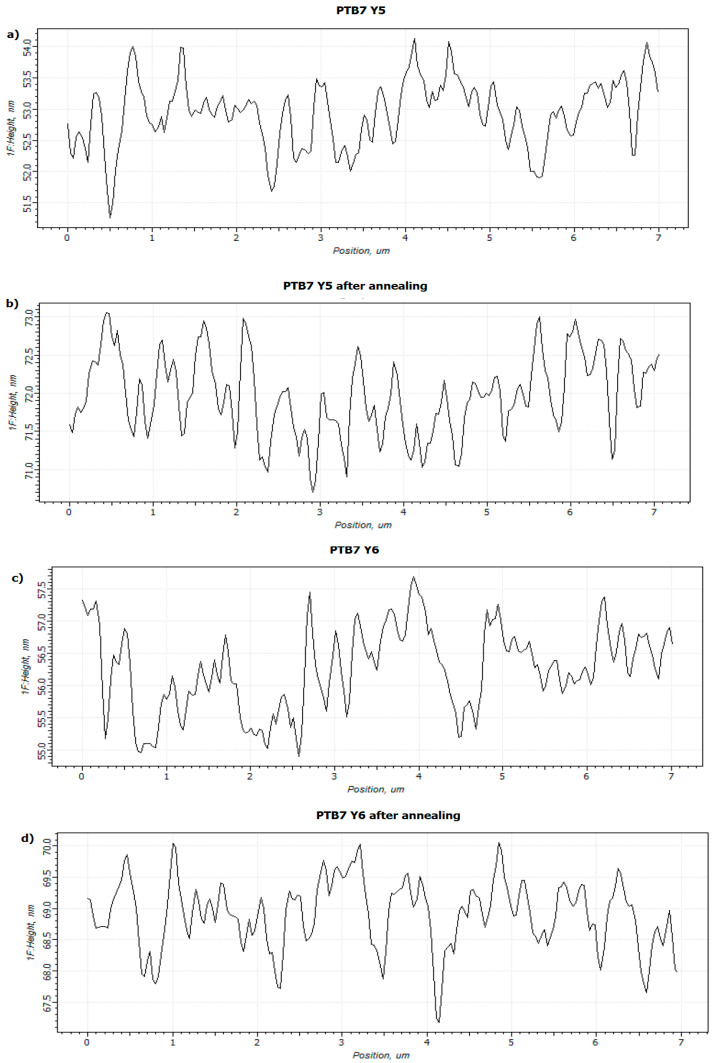
AFM height profile for (**a**) PTB7 Y5 before heating, (**b**) PTB7 Y5 after annealing, and (**c**) PTB7 Y6 before and (**d**) after annealing.

AFM studies on binary mixture thin films were conducted, and the results can be found in Figure 16. The PTB7:Y5 film has a fairly smooth surface with uneven features or pits. Appendix A includes the AFM profiles (Appendix A, Figure A3). After annealing, the surface appears more uniform, with reduced roughness and fewer pits.

The roughness of the layers changes slightly. For PTB7:Y5, Rq changes from 2.6 nm to 3.0 nm after annealing, and Ra changes from 1.0 nm to 0.96 nm. In contrast, the PTB7:Y6 thin film roughness Rq is 3.3 nm, the Ra is 1.2 nm before annealing, and after annealing, the Rq is 3.7 nm, and the Ra is 1.4 nm.

Gao et al. measured Y6 blended with poly(3-hexylthiophene) (P3HT), which is also a thiophene-based donor, in organic solar cells. These studies imply its crystallization behavior and thus moderate thermal stability [58]. Its susceptibility to temperature-induced crystallization and aggregation leads to undesirable morphological changes in the blend and decreased device performance.

Compared with pure acceptor films, blending PTB7 with Y5 or Y6 generally enhances the thermal stability, with the resulting films maintaining relatively stable optical properties even after heating. The morphology remains largely unchanged, with minor variations in roughness.

### 3.4. Binary Mixtures Based on PTB7th Donor Thermal Stability

**Figure 17:**
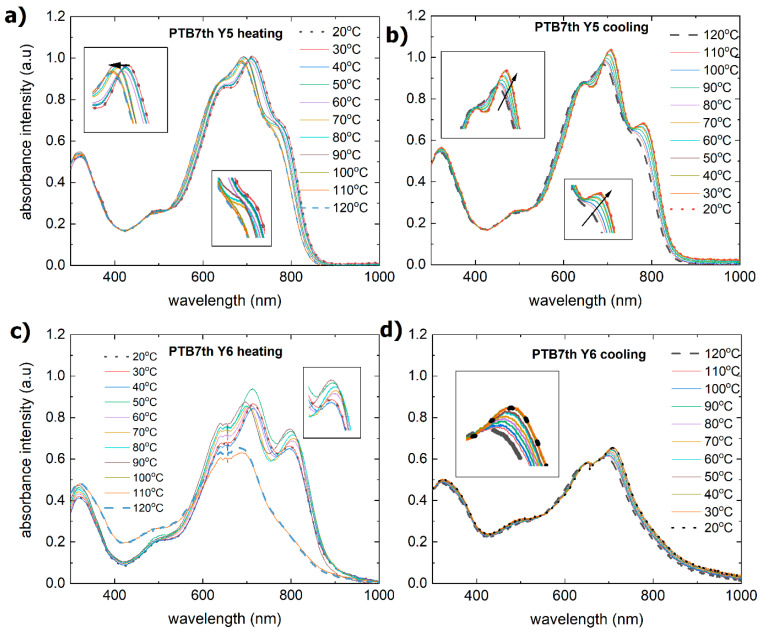
Absorbance spectra of the investigated binary thin films: (**a**) PTB7th:Y5 during heating, (**b**) PTB7th:Y5 during cooling, (**c**) PTB7th:Y6 during heating, and (**d**) PTB7th:Y6 during cooling. Arrows on the figure indicate the direction of changes.

The absorption spectrum of PTB7th:Y5 (Figure 17) is broad, with peaks at 500 nm, 610 nm, 700 nm, and 790 nm. During heating, the maxima at 705 nm and 785 nm decrease in intensity and shift toward short wavelengths. The absorption spectrum of PTB7th:Y6 shows absorption maxima at 500 nm and more pronounced maxima at 620 nm, 710 nm, and 800 nm.

Above 100 °C for PTB7th:Y6, there was a decrease in the absorption intensity, and the peak at 800 nm disappeared. During cooling, the intensity is stable, and the peak does not reappear, so the temperature changes are permanent. The decrease and disappearance of the peak for PTB7th:Y6 is related to the decrease in the absorption intensity of Y6, which is observed earlier and does not return to its initial state.

**Figure 18:**
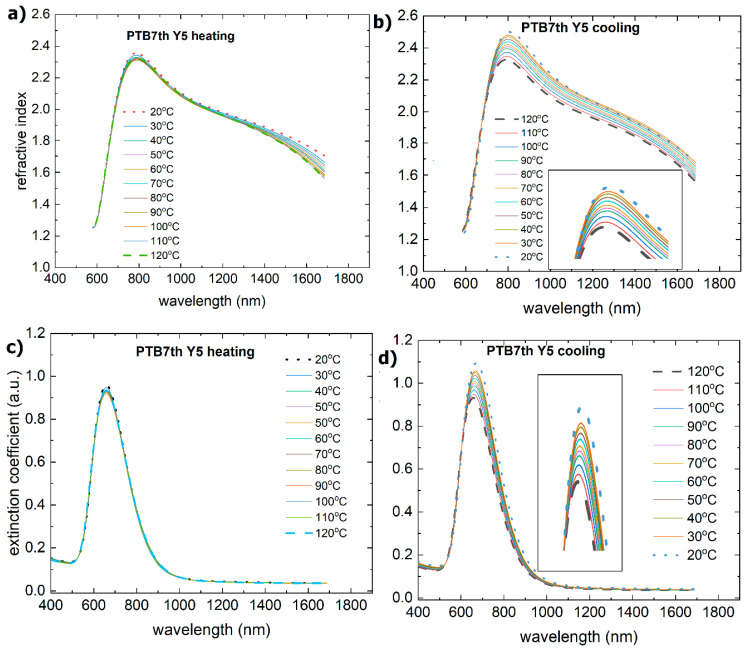
Temperature changes as a function of wavelength for the (**a**) refractive index (heating), (**b**) refractive index (cooling), (**c**) extinction coefficient (heating), and (**d**) extinction coefficient (cooling) for the PTB7th:Y5 mixture.

The thin film PTB7th:Y5 during heating exhibited changes in the dispersion dependence of the refractive index and extinction coefficient, as shown in Figure 18. During cooling, there is a slight increase in these quantities from 600 nm.

**Figure 19:**
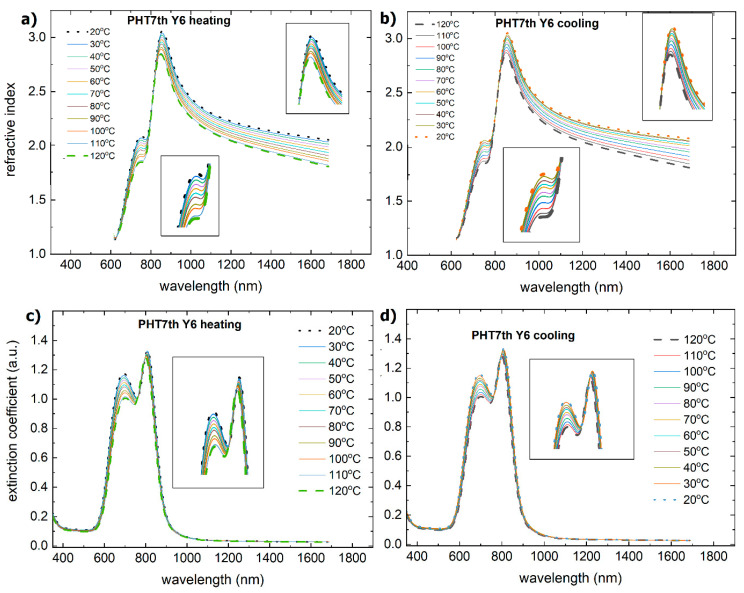
Temperature changes as a function of wavelength for (**a**) the refractive index (heating), (**b**) the refractive index (cooling), (**c**) the extinction coefficient (heating), and (**d**) the extinction coefficient (cooling) for the PTB7th:Y6 mixture.

**Figure 20:**
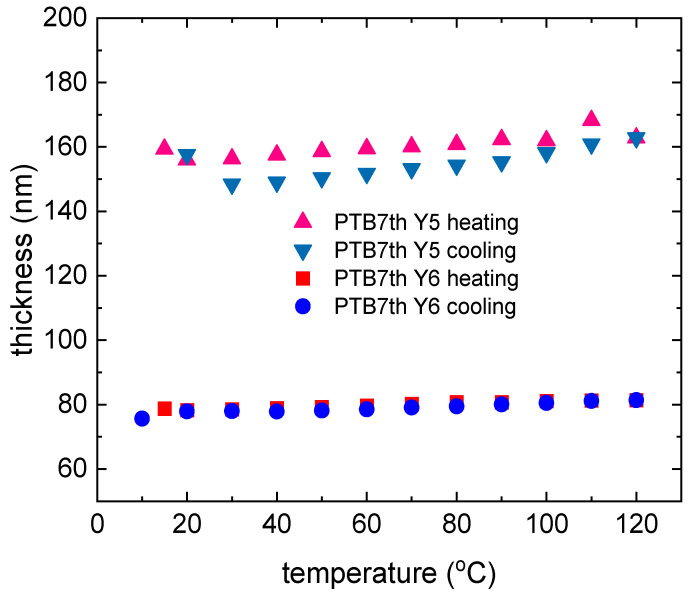
Thin film thickness dependence of the PTB7th:Y5 and PTB7th:Y6 thin film mixtures during the heating and cooling processes.

The dispersion relationships of the refractive indices for the PTB7th:Y6 thin films show changes in the 650–700 nm and above 820 nm regions (Figure 19). The extinction coefficient shows sensitivity to temperature changes only for a maximum of 620 nm; in the case of heating, it decreases, and then, with cooling, it increases. The changes are reversible for both the refractive index and extinction coefficient. The thin film thickness varies from a few nanometers for PTB7th:Y5 (Figure 20). In the case of the PTB7th:Y6 layer, essentially no changes are observed; this layer is the most stable of those tested. The symbol’s size is regarded as the degree of uncertainty in determining its thickness.

**Figure 21:**
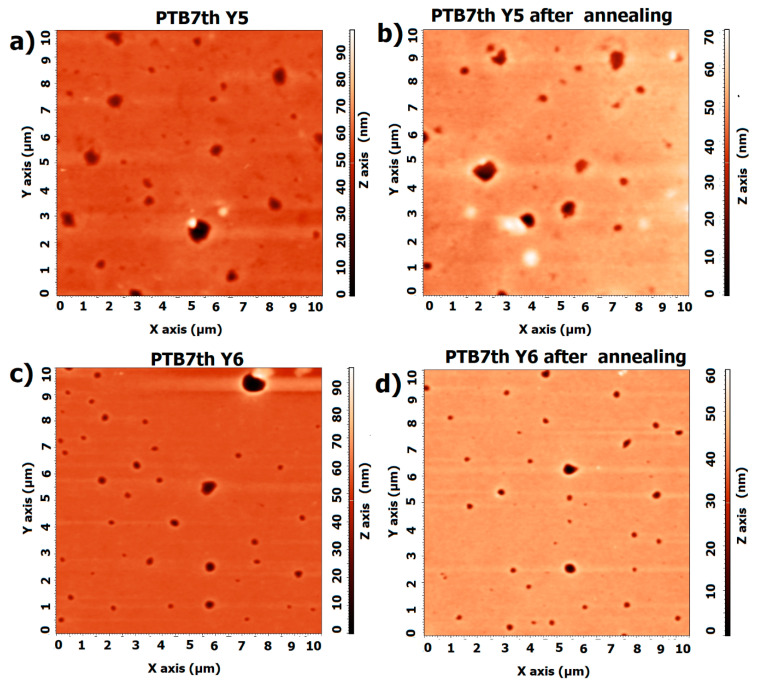
AFM images of thin films (**a**) PTB7th:Y5 before annealing, (**b**) PTB7th:Y5 after annealing, (**c**) PTB7th:Y6 before annealing, and (**d**) PTB7th:Y6 after annealing.

The AFM images of both thin films PTB7th:Y5 and PTB7th:Y6 (Figure 21) show a relatively smooth surface with a few noticeable features or pits. The surface roughness appears to be low, indicating a fairly uniform film. Post-annealing, the surface seems to have similar features, but the overall topography looks slightly smoother with possibly fewer defects, such as pits. Annealing often helps improve uniformity by reducing surface roughness [62,63,64]. No phase changes or changes in the form of the layer are observed.

**Figure A4:**
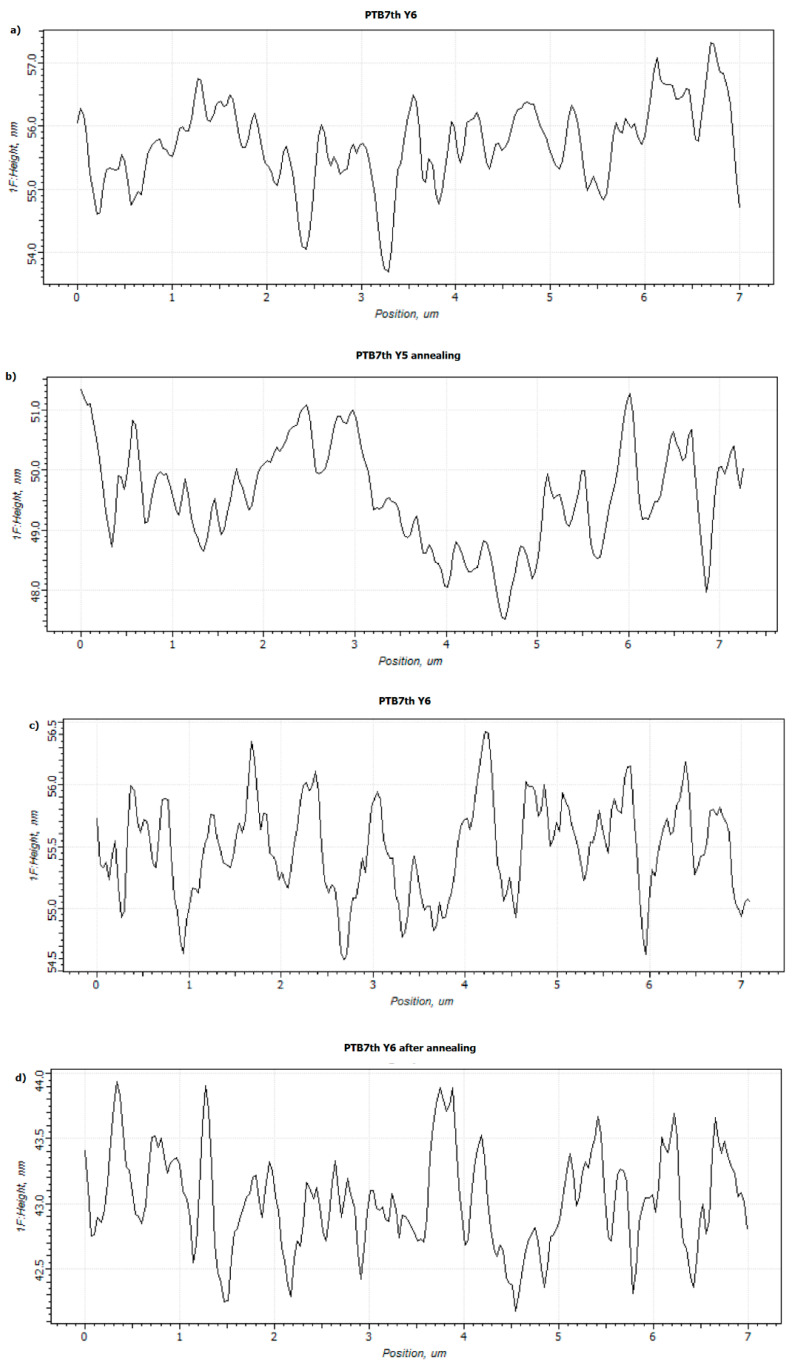
AFM height profile for (**a**) PTB7th Y5 before heating, (**b**) PTB7th Y5 after annealing, and (**c**) PTB7th Y6 before and (**d**) after annealing.

For the PTB7th:Y5 film, the AFM measurements show that Rq changes from 4.1 nm to 4.0 nm, and Ra changes from 1.8 nm to 1.9 nm, whereas the PTB7Tth:Y6 layer reduces roughness during annealing from Rq 4.0 nm to 2.3 nm and Ra from 1.3 nm to 0.85 nm. The AFM profiles are shown in Appendix A (Figure A4).

The pure donor layers showed variable layer thicknesses, the morphology studied with AFM was unchanged, and small changes in the dispersion relationships of the refractive and extinction coefficients were also observed. The features of NFA layers Y5 and Y6 are not very consistent; however, changes in the absorption spectrum were confirmed; however, in the case of Y5, changes in the dispersion dependence of the refractive index and extinction coefficient were observed. NFA, in terms of optical properties, is less stable; however, the thin layer has a constant thickness.

The optical properties of the donor–acceptor layers based on PTB7 are stable, as confirmed by absorption studies, the weakly variable dispersion relationships of the refractive and extinction coefficients, and the constant morphology. In this case, however, small changes in the film thickness are observed. Layers based on PTB7th are less stable. The absorbance of the PTB7thY6 layer was similar to that of Y6. The PTB7th:Y5 layer shows a thickness variation similar to that of pure PTB7th. In the PTB7th:Y6 layer, the thickness is as stable as that of Y6. Thus, in different mixtures, the properties of different components dominate. The donors sufficiently stabilize the morphologies of both the PTB7th:Y5 and PTB7th:Y6 layers so that no phase change is observed (it is not visible via AFM). Annealing appears to decrease surface roughness and may improve uniformity for PTB7:Y5 and PTB7:Y6, which is advantageous for applications requiring films free of defects.

PTB7th and the non-fullerene acceptors Y5 and Y6 interact at the molecular level through various forces (π–π stacking) [65,66]. These interactions affect the blend’s overall electronic structure, energy levels, and molecule packing. The nature and strength of these interactions are affected by temperature. The various observed changes are a result of the different interaction intensities between PTB7th and Y5 as opposed to Y6. At elevated temperatures, the molecular mobility of both the donor and acceptor molecules increases.

The reversible change in thickness with temperature, while not directly beneficial in itself, provides valuable information for optimizing photovoltaic cell design and performance. It is not a feature actively utilized in the cell’s operation, but rather a characteristic that helps understand and improve several aspects of solar cells’ stability. Uncertainties of thickness measurement are around nanometers, depending on the sample, and are mainly related to the uncertainty of fitting the ellipsometric model.

High temperatures can lead to the thermal degradation or decomposition of organic materials, resulting in the loss of functional groups or the complete breakdown of the molecular structure, which can diminish absorption. It can also cause conformational changes within the molecular structures of organic materials, which can affect the electronic transitions responsible for light absorption. In systems of binary mixtures, the stability is increased, as demonstrated by Paleti for five structurally similar acceptors, improving the thermal stability of non-fullerene-based organic solar cells [67]. Variations can also be caused by the occurrence of a matrix effect.

The donor polymers PTB7 and PTB7th show reversible changes in thickness and absorption with temperature, but the extent of these changes differs between PTB7 and PTB7th, indicating sensitivity to subtle structural differences. Non-fullerene acceptors (Y5, Y6) exhibit different thermal behaviors. Y5 shows some reversible changes, while Y6 demonstrates greater instability and more permanent changes at higher temperatures. The most important finding is likely the observed enhanced thermal stability of binary mixtures compared to single-component materials. Blending donor and acceptor materials appears to mitigate some of the negative thermal effects observed in individual components. The PTB7-based blends show particularly promising stability.

The matrix effect results from intermolecular interactions that occur between the molecules of a substance and the molecules of its environment. These interactions can affect the electron energies of the absorbing molecule, resulting in a change in its absorption [68,69]. Organic thin films can undergo phase transitions with increasing temperature, which can alter the arrangement of molecules and reduce the absorption efficiency [70,71,72]. This work has focused on optical issues; however, electron and hole mobility is also an essential issue in the context of organic cells. As previous studies by Ebenhoch et al. have shown [73], for PTB7:PC71BM cooled from ambient temperature to 77 K, the temperature dependence showed a rapid decline in hole mobility of three orders of magnitude; moreover, at low mobility, PTB7:PC71BM cells’ solar cell performance declines. The research by Tang et al. [74] showed bimolecular recombination as the dominant recombination mechanism, and the relationship of open circular voltage with temperature was determined for PTB7-Th:PC71BM bulk-heterojunction organic solar cells in the range of 160–295 K. The studies by Lee at al. indicated a decrease in performance of cells with ITO/HEL/P3HT: PCBM/LiF/Alo architecture of 20% with temperature (near 300–420) [75].

## 4. Summary

This research describes the optothermal behavior of single- and binary-component layers, which is crucial for organic photovoltaic cells. This study focuses on how temperature affects the morphology and optical characteristics of thin films constructed of materials that are frequently employed in these kinds of devices, specifically PTB7 and PTB7th as donors and Y5 and Y6 as non-fullerene acceptors. The results showed that the optical properties and morphology of the thin films are sensitive to temperature changes. From the perspective of applications as an active layer in organic cells, the observed changes affect device performance. To obtain further conclusions, it would be necessary to examine the electrical properties as well; however, the thermo-optical properties are satisfactory. Further improvements in performance and stability can be achieved by blending additional materials [76,77]. This study highlights the importance of controlling the morphology evolution in organic solar cells to achieve stable and efficient devices. The authors emphasize the need for further research on the relationships between temperature, morphology, and device performance.

## Data Availability

Data sets generated during the current study are available from the corresponding author upon reasonable request.
